# Four decades of data indicate that planted mangroves stored up to 75% of the carbon stocks found in intact mature stands

**DOI:** 10.1126/sciadv.adk5430

**Published:** 2024-07-05

**Authors:** Carine F. Bourgeois, Richard A. MacKenzie, Sahadev Sharma, Rupesh K. Bhomia, Nels G. Johnson, Andre S. Rovai, Thomas A. Worthington, Ken W. Krauss, Kangkuso Analuddin, Jacob J. Bukoski, Jose Alan Castillo, Angie Elwin, Leah Glass, Tim C. Jennerjahn, Mwita M. Mangora, Cyril Marchand, Michael J. Osland, Ismaël A. Ratefinjanahary, Raghab Ray, Severino G. Salmo, Sigit D. Sasmito, Rempei Suwa, Pham Hong Tinh, Carl C. Trettin

**Affiliations:** ^1^ Institute of Pacific Islands Forestry, Pacific Southwest Research Station, USDA Forest Service, Hilo, HI 96720, USA.; ^2^ Institute of Ocean and Earth Sciences, University of Malaya, Kuala Lumpur 50603, Malaysia.; ^3^ Center for International Forestry Research (CIFOR), International Centre for Research in Agroforestry (ICRAF); D. P. Wijesinghe Mawatha, Battaramulla, Colombo, Sri Lanka.; ^4^ Institute of Pacific Islands Forestry, Pacific Southwest Research Station, USDA Forest Service, Albany, CA 94710, USA.; ^5^ US Army Engineer Research and Development Center, Vicksburg, MS 30180, USA.; ^6^ Department of Oceanography and Coastal Sciences, Louisiana State University, Baton Rouge, LA 70803, USA.; ^7^ Conservation Science Group, Department of Zoology, University of Cambridge, Cambridge CB2 3QZ, UK.; ^8^ US Geological Survey, Wetland and Aquatic Research Center, Lafayette, LA 70506, USA.; ^9^ Biotechnology Program, Mathematics and Natural Sciences, Universitas Halu Oleo, Kendari, Southeast Sulawesi 93232, Indonesia.; ^10^ Department of Forest Ecosystems and Society, College of Forestry, Oregon State University, Corvallis, OR 97331, USA.; ^11^ Ecosystems Research and Development Bureau, Department of Environment and Natural Resources, Forestry Campus, Los Baños 4031, Philippines.; ^12^ Department of Geography and Environmental Science, University of Reading, Reading RG6 6AB, UK.; ^13^ Blue Ventures Conservation, Antananarivo 101, Madagascar.; ^14^ Leibniz Centre for Tropical Marine Research, Bremen 28359, Germany.; ^15^ Faculty of Geosciences, University of Bremen, Bremen 28359, Germany.; ^16^ Institute of Marine Sciences, University of Dar es Salaam, Buyu Campus, Zanzibar P.O. Box 668, Tanzania.; ^17^ ISEA, Université de la Nouvelle-Calédonie, Nouméa, New Caledonia 98851, France.; ^18^ Atmosphere and Ocean Research Institute, The University of Tokyo, Kashiwa 277-8564, Japan.; ^19^ Institute of Biology, College of Science, University of the Philippines Diliman, Quezon City 1101 Philippines.; ^20^ NUS Environmental Research Institute, National University of Singapore, Singapore 117411, Singapore.; ^21^ Japan International Research Center for Agricultural Sciences (JIRCAS), Tsukuba 305-8686, Japan.; ^22^ Faculty of Environment, Hanoi University of Natural Resources and Environment, Hanoi 10000, Vietnam.; ^23^ Center for Forested Wetlands Research, Southern Research Station, USDA Forest Service, Cordesville, SC 29434, USA.

## Abstract

Mangroves’ ability to store carbon (C) has long been recognized, but little is known about whether planted mangroves can store C as efficiently as naturally established (i.e., intact) stands and in which time frame. Through Bayesian logistic models compiled from 40 years of data and built from 684 planted mangrove stands worldwide, we found that biomass C stock culminated at 71 to 73% to that of intact stands ~20 years after planting. Furthermore, prioritizing mixed-species planting including *Rhizophora* spp. would maximize C accumulation within the biomass compared to monospecific planting. Despite a 25% increase in the first 5 years following planting, no notable change was observed in the soil C stocks thereafter, which remains at a constant value of 75% to that of intact soil C stock, suggesting that planting effectively prevents further C losses due to land use change. These results have strong implications for mangrove restoration planning and serve as a baseline for future C buildup assessments.

## Teaser

C stock in planted mangroves plateaued at 75% of intact stands after 20 years with no soil changes 5 years post-planting onward.

## INTRODUCTION

In conjunction with historical losses, an estimated 35% of global mangrove area has been lost over the past five decades to human-driven land-use change, extreme weather events, and erosion (*1*–*3*). However, growing awareness around mangrove-dependent socio-ecological well-being has led to important conservation and restoration efforts of these ecosystems, with annual deforestation rates declining from 0.7 to 1% in the 1980s to 1990s to 0.2 to 0.4% in the early 2000s (*1*, *4*). Because mangroves have one of the highest net ecosystem productivity rates and carbon (C) storage potential on the globe (*5*–*7*), restoring or rehabilitating these ecosystems has been regarded as a promising long-term nature-based solution to partly offset emissions of greenhouse gases (GHGs) while simultaneously enhancing biodiversity and contributing to coastal protection (*8*, *9*).

Although research is increasingly highlighting the greater suitability of (assisted) natural regeneration and hydrological restoration, planting remains the predominant mangrove restoration and rehabilitation strategy, despite the fact that many planting attempts fail, largely due to planting species in unsuitable biophysical conditions (*10*, *11*). Despite the perceived benefit of restoration, there is now no consensus on the timeline required for successful planted mangrove stands to recover or build up levels of C stocks similar to natural mangrove forests, with alluded periods ranging anywhere from 20 to 50 years (*12*–*18*) to over a century (*19*). As the United Nation (UN) general assembly has declared 2021 to 2030 as the UN Decade on Ecosystem Restoration (*20*), mangrove restorable area is estimated at 8120 km^2^, of which 6665 km^2^ are considered to be highly restorable (*21*). Understanding how effective past mangrove restoration projects have been at returning antecedent C stocks across different locations and species composition is therefore critical in prioritizing future efforts and maximizing success in these restorable areas.

Here, we assessed whether mangrove planted stands demonstrate similar ability to store C as natural primary stands including primary forests including intact forest landscapes (PF-IFL), i.e., free of notable human degradation (*22*), hereafter called “intact,” as well as within which timelines. Briefly, we collected 40 years of data on C stocks in planted stands, including in restored/rehabilitated (i.e., where mangroves were previously present but had been degraded or entirely deforested) and afforested (where there was no known record of mangroves before planting) mangrove stands worldwide varying in species composition. Using logistic growth models implemented in a Bayesian generalized nonlinear modelling framework (see Materials and Methods), we then quantified the C stock buildup ratio, *R*, of these restored or afforested mangroves relative to intact stands in the vicinity.

## RESULTS

### Global patterns

Our database consists of aboveground biomass (AGB), belowground biomass (BGB), and soil C stock (down to 1-m depth) data collated from 809 restored and afforested mangrove stands (hereafter referred to as “planted” stands) distributed across 24 countries and 181 geomorphic sites (i.e., in a particular estuary, delta, open-coast area, or lagoon). We also collected data from 475 intact stands distributed across 185 sites located near these planted stands (see Materials and Methods).

Most of the planted stands recorded in this study were monospecific (*n* = 670) as opposed to mixed-species (*n* = 139). The most common genus recorded in monoculture stands was *Rhizophora* (*n* = 346), followed by *Avicennia* (*n* = 98), *Sonneratia* (*n* = 94), and *Kandelia* (*n* = 69). A vast geographic disparity existed between the number of published data on planted stands located in the Atlantic-East Pacific region (14% of the data) and in the Indo-West Pacific region (86% of the data), with the Indo-Malaysian continental region alone accounting for 77% of total observations (fig. S1).

For all stand ages and in each region, soil C stock (down to 1-m depth) represented the majority of the total C reservoir in planted mangroves, followed by the aboveground and then belowground C stock within the tree biomass. The soil C stock proportion peaked at 83 to 95% of the total ecosystem C stock in the first 5 years after planting (see table S1). As the aboveground and belowground C stocks within the tree biomass increased steadily with time, this proportion decreased to 45 to 50% 35 years after planting.

**Fig. 1.**
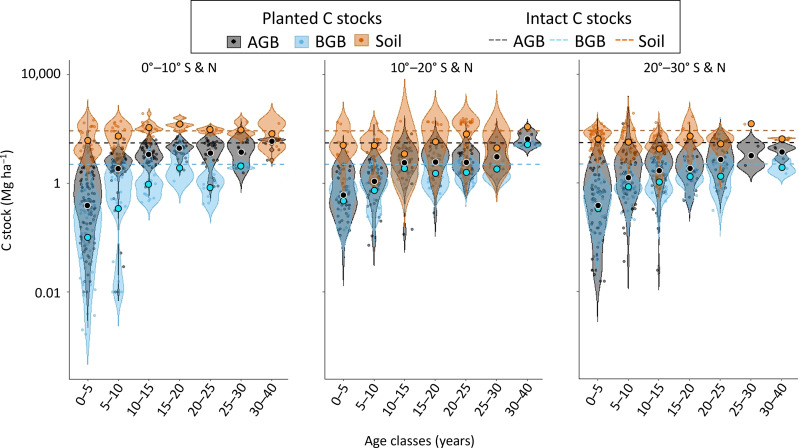
Visual distribution of planted mangrove C stock data over time and latitude zones. Violin plots of AGB (gray), BGB (blue), and soil (orange) C stocks (megagrams of carbon per hectare, logarithmic scale) data binned across absolute latitudinal zones and age classes for the planted mangroves. The mean C stock values in planted stands are indicated by larger filled circles, and the density distribution of observations is indicated by smaller circles and respective outlining beams. Dashed lines indicate the mean global values found in the literature for intact mangrove forests for comparison, i.e., mean intact AGB C stock = 99.05 Mg ha^−1^ (*55*); mean intact BGB C stock = 48.91 Mg ha^−1^ (data S1); mean intact soil C stocks down to 1 m = 276.65 Mg ha^−1^ (*56*).

The highest soil and aboveground C stocks were found between 0° and 10° N and S, whereas the highest belowground C stocks were found between 10° and 20° N and S. Nevertheless, the influence of latitude on the soil, BGB, and AGB C stock variations was not significant after adjusting for planted stand age (probability of analysis of covariance > 0.05). However, some age classes remain widely underrepresented (Fig. 1).

### C stock variations in planted mangroves: Nonlinear logistic growth models

**Fig. 2.**
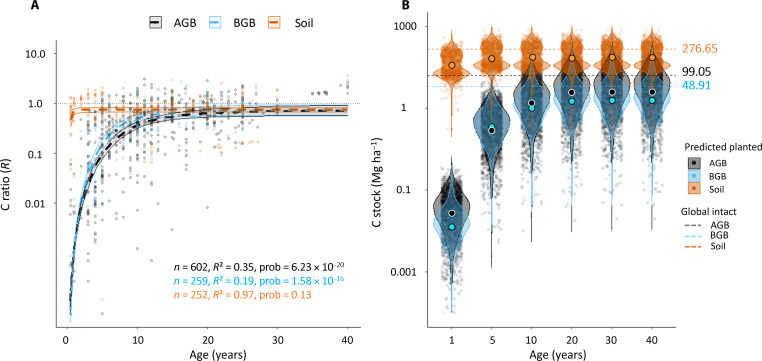
Logistic growth models of the C stock buildup ratios *R* over time in planted mangroves relative to intact mangrove stands, and visualization of the worldwide C stock buildup values (*R*) predicted by these models. (**A**) Comparative view of the C stock buildup ratio (*R*) logistic growth models over time in the AGB (gray), BGB (blue), and soil (orange) in planted mangroves (logarithmic scale for the C stock buildup ratio axis). For each model, the number of observations (*n*), coefficient of determination (*R*^2^), and the probability (prob) are indicated; (**B**) violin plots of the worldwide predicted C stock buildup (megagrams per hectare, logarithmic scale) in the AGB, BGB, and soil at years 1, 5, 10, 20, 30, and 40 following planting. Mean predicted C stocks are indicated by large circles, while the mean global intact C stocks are indicated by dashed lines for comparison. The predicted C stocks were calculated by multiplying the ratios *R* at age 1, 5, 10, 20, 30, and 40 years following planting to each intact mangrove C stock data found in the literature for the AGB [*n* = 2 709; (*55*)], BGB (*n* = 340, data S1), and soil [*n* = 1239; (*56*)] compartments.

Overall, both BGB and AGB C stock buildup ratios (*R*) in planted mangroves increased sharply over the first 20 years. The belowground C stock buildup ratios increased from *R* = 0.001 at time 0 to 0.70 [95% confidence interval (CI) = 0.56 to 0.88] at 20 years, whereas the aboveground C stock ratios increased from 0.00001 at time 0 to 0.63 (95% CI = 0.53 to 0.76) at 20 years (Fig. 2A, probability < 0.05). Subsequently, the slope rise became more gradual, until reaching an *R*_max_ of 0.73 (95% CI = 0.58 to 0.94) for BGB and 0.71 (95% CI = 0.57 to 0.91) for AGB at 40 years. Notably, belowground C stock ratios tended to build up faster than aboveground C stock buildup ratios in the earlier years (0 to 15 years) of planting.

In contrast to the belowground and aboveground C stocks, the mean soil C stock value at the time of planting (*t* = 0) was about half that of intact mangrove stands (95% CI *R* = 0.33 to 0.73). Within the five first years following planting, the mean soil C stock buildup ratio *R* increased from 0.48 to 0.74. However, we observed stronger variations in the *R* values during that period (0 to 5 years) than during the rest of the studied period (5 to 40 years), with mean *R* value ± SD at time 0 = 0.48 ± 0.34 (range, 0.05 to 1.32; *n* = 22) and mean *R* value ± SD = 0.86 ± 0.46 (range, 0.05 to 2.06; *n* = 95) for the five first years following planting. Following this initial period, the mean soil C stock buildup ratio *R* remains at a constant value of 0.75 from 5 to 40 years following planting, and the resulting logistic growth curve accounts for most of the variations in our soil C stock buildup ratio dataset [coefficient of determination (*R*^2^) = 0.97]. Overall, evidence from our model suggested a low variation of soil C stock in planted stands relative to intact stands (slope not notably different from 0, posterior probability = 0.13).

We then used these logistic growth models to predict the global ecosystem C stocks in planted stands at a chosen time after planting by multiplying the buildup ratios *R* found in our models at a given time *t* by each intact mangrove C stock data found in the literature for each of the ecosystem compartments. When subtracting the predicted values found at 40 years by the predicted values found at time of planting (*t* = 0), we found that these C stock increases equate to a mean net ecosystem C gain of 143.21 MgC ha^−1^ after 40 years (95% CI = 83.03 to 182.86 MgC ha^−1^) (Fig. 2B and table S2).

**Fig. 3.**
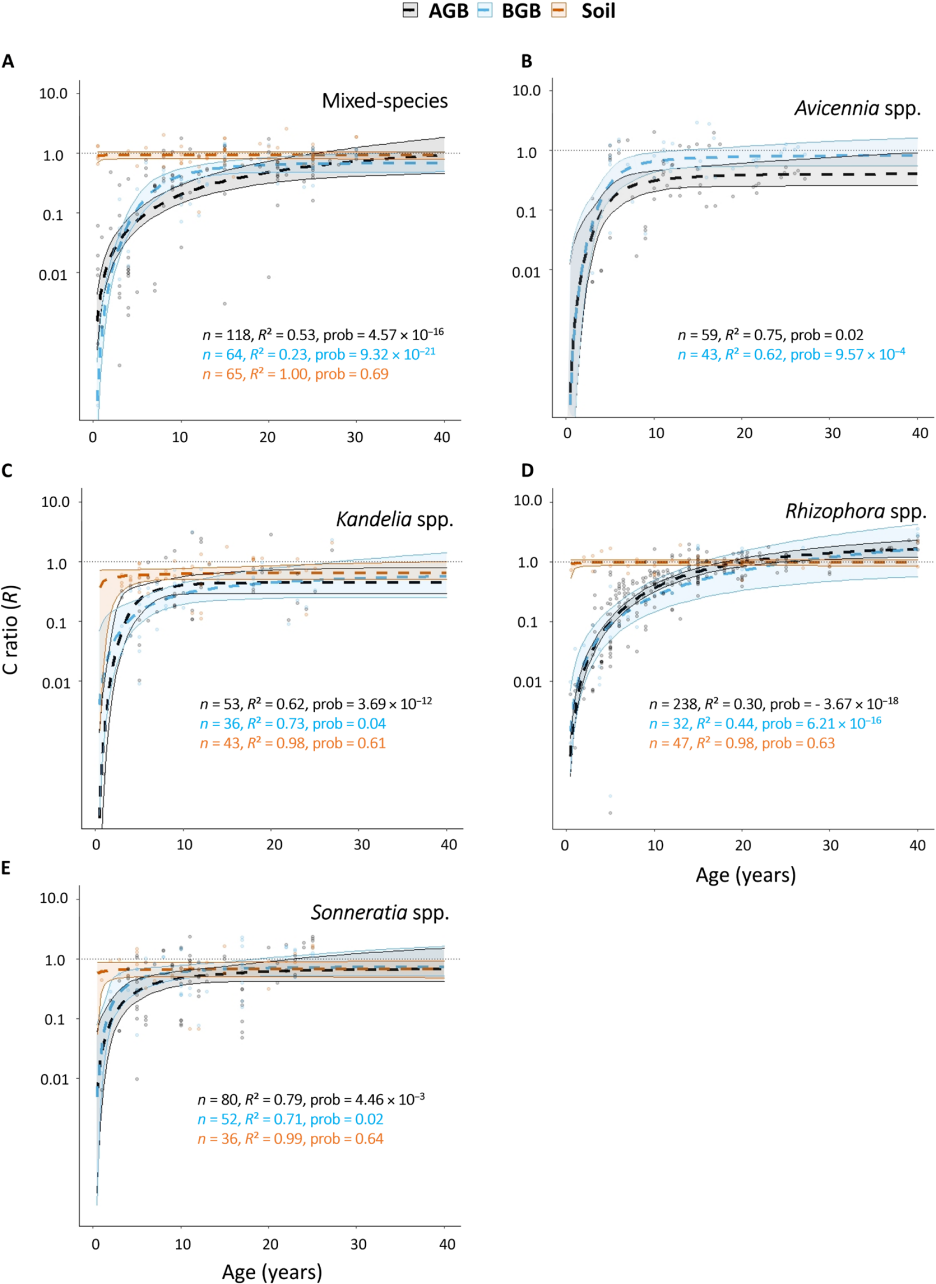
Logistic growth models of the C stock buildup ratios *R* over time in the different genera and mixed-species mangrove planted stands relative to intact mangrove stands, and visualization of the worldwide C stock buildup values *R* predicted by these models. (**A** to **E**) C stock ratio logistic growth models over time in the AGB (gray), BGB (blue), and soil (orange) in mixed-species, *Avicennia*, *Kandelia*, *Rhizophora*, and *Sonneratia* planted stands (logarithmic scale for the C stock ratio axis). For each model, the number of observations *n*, coefficient of determination *R*^2^, and the probability are indicated.

**Fig. 4.**
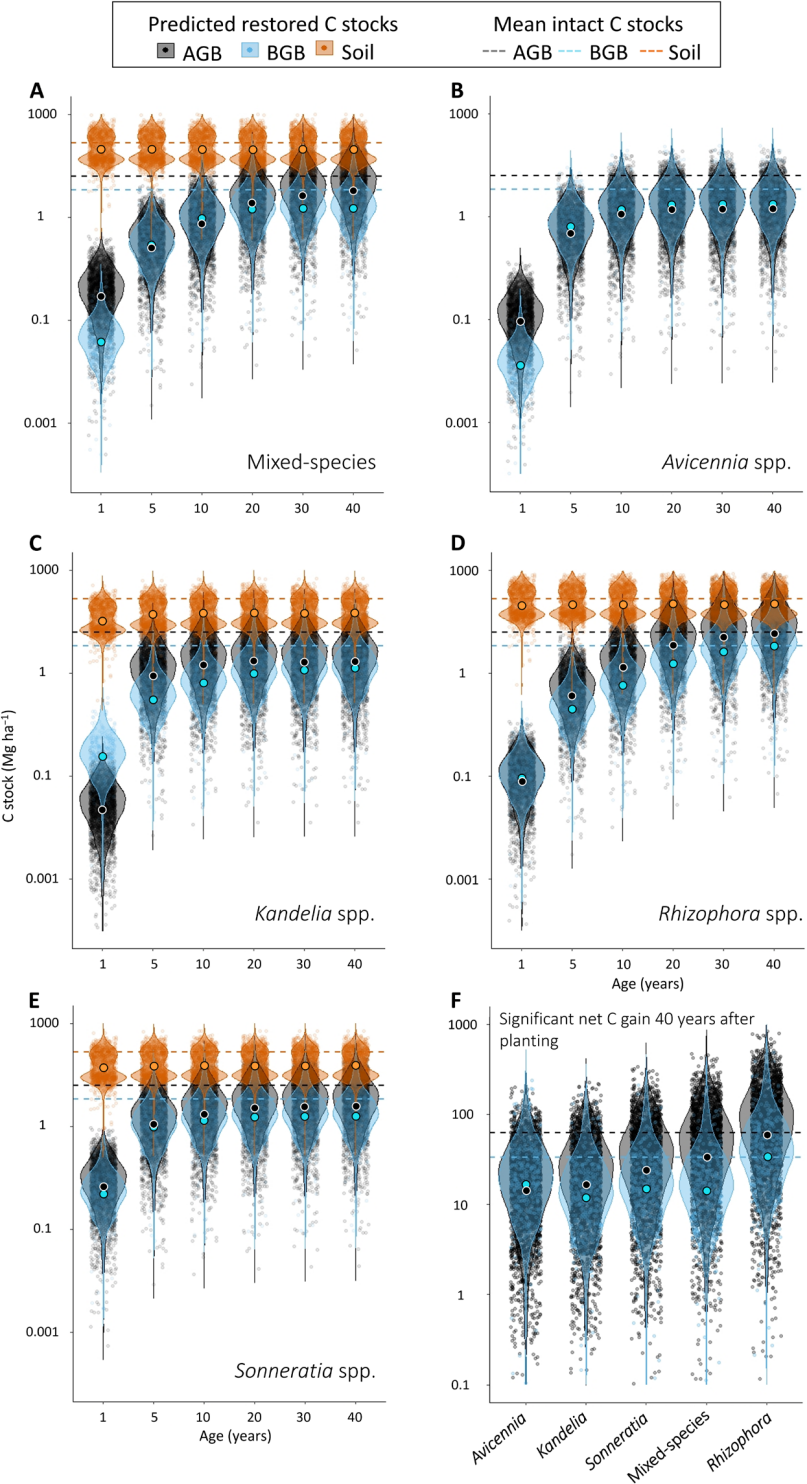
Comparative visualization of the worldwide C stock buildup in mangroves planted stands with the most common genera and mixed-species predicted by the logistic growth models. (**A** to **E**) Multispecies and genus-specific violin plots of the worldwide predicted C stock buildup in the AGB, BGB, and soil 1, 5, 10, 20, 30, and 40 years after planting; mean predicted C stocks are indicated by large circles, while the mean global intact C stocks are indicated by dashed lines (**F**) comparison of the predicted net C stock gain built up worldwide 40 years after planting for each genus and mixed-species planted stands. For all figures, the predicted C stocks were calculated by multiplying the ratios *R* compiled for each genus and mixed-species planted stands at age 1, 5, 10, 20, 30, and 40 after planting by each intact mangrove C stock data found in the literature for the AGB [*n* = 2709; (*55*)], BGB (*n* = 340, data S1), and soil [*n* = 1239; (*56*)] compartments.

The logistic growth model slopes of the mixed-species and different genera did not reveal any notable variations of the soil C stock buildup ratios over time (i.e., slopes not significantly different from 0, probabilities > 0.50; Fig. 3). In addition, there was no significant difference between the logistic growth model slopes of the mixed-species and the different genera, suggesting that there was no effect of diversity on the soil C stock buildup ratio variation in the studied period (probabilities > 0.50). On the other hand, C stock buildup within the biomass was generally higher in mixed-species planted stands when compared to monospecific planted stands, with the exception of *Rhizophora* planted stands (probabilities < 0.05). The latter were able to store up to 1.42 times more C in their biomass (95% CI *R*_max_ = 1.20 to 2.28 and 0.56 to 4.23 for BGB and AGB, respectively) than intact forests after 40 years, followed by mixed-species planted stands (95% CI *R*_max_ = 0.45 to 1.81 and 0.48 to 1.03), and then monospecific *Sonneratia* (95% CI *R*_max_ = 0.42 to 1.50 and 0.51 to 1.62), *Avicennia* (95% CI *R*_max_ = 0.25 to 0.90 and 0.54 to 1.59) and *Kandelia* (95% CI *R*_max_ = 0.29 to 0.79 and 0.25 to 1.40) planted stands (Figs. 3 and 4 and table S2). During the first 10 years after planting, *Sonneratia* and *Avicennia* presented higher C stock buildup ratios in their BGB, with mean *R* values of 0.65 (95% CI = 0.46 to 0.79) and 0.61 (95% CI = 0.42 to 0.94), respectively, compared to 0.28 (95% CI = 0.19 to 0.45), 0.24 (95% CI = 0.14 to 0.51), and 0.41 (95% CI = 0.30 to 0.54) after the same period of time for *Kandelia*, *Rhizophora*, and mixed-species planted stands, respectively (Fig. 3).

## DISCUSSION

This meta-analysis aims to assess the ability of mangrove planted stands in returning C stocks up to values similar to that of intact primary mangrove forests. Our analysis of 40 years of data on C stocks in planted mangrove forests shows that, on average, C stock values in planted stands reached ~75% of the ecosystem C stocks measured in intact mangrove stands (table S2). However, our models also indicate that the increase in C stock in planted stands relative to intact stands is only significant with time within AGB and BGB, in which biomass culminated at 71 and 73% after 40 years, respectively, with ~90% of this accumulation occurred over the first 20 years of planting. This corresponds to a net C accumulation of 69.13 Mg ha^−1^ (55.34 to 88.8 Mg ha^−1^, BGB and AGB combined). On the basis of recent global estimates and assuming a similar success in the future, the reforestation of 6665 km^2^ of highly restorable mangroves (*21*) has a potential to store close to 46 × 10^06^ MgC in mangrove biomass alone in 20 years. This equates to annual fossil fuel emissions from nearly the entire United Kingdom road motor vehicles circulating in 2022 (*23*) or accounts for 0.025% of global annual CO_2_ emissions [37.25 × 10^09^ MgCO_2_ in 2021; (*24*)].

Conversely, mean soil C stock value in planted mangroves at time of planting was only half of what was found in intact mangrove stands. Despite a ~25% increase in the first 5 years following planting (95% CI of the net C gain = 27.69 to 94.06 Mg ha^−1^), no notable fluctuation in the soil C stock ratio was found thereafter, with a consistent mean C stock buildup ratio *R* value of 0.75 between 5 and 40 years following planting (*R*^2^ = 0.97, posterior probability = 0.13). This lack of evidence for an increase in C stock in planted stands relative to intact stands over time despite the initial increase of ~25% could simply reflect the diverse heterogeneity-generating processes at local spatial scales (e.g., geophysical factors and hydrographic processes along the intertidal gradient) (*25*), which were not captured by the inter-site comparison approach chosen here. Furthermore, previous land cover and land use, as well as the adopted planting strategy—including the growing conditions (nursery or direct seeding), planting density, and spacing—are among the various key factors that could influence C accumulation in the standing biomass and soils of planted mangrove stands (*26*). These factors could explain the high variations observed in our model during the first 5 years following planting. While increases in soil C concentration were reported with forest age in several studies, a review shows that this increase in C stock was particularly notable in the initial years (0 to 5) of forest growth, with wide variations across study sites. This increase notably decreased thereafter, aligning closely with our own observations [with *n* = 31; (*27*)]. Except for the increase in C stock during the initial years following planting, our findings, indicating a consistent *R* ratio value of 0.75 from 5 to 40 years, suggest that the 40 years’ time frame covered by our observations is too short to observe any additional storage of recalcitrant C in planted mangrove soils relative to intact stands at a global scale, all the more so in an environment prone to tidal export. We emphasize that a limitation of our model is that it only accounts for net soil C stock variations over time, without specifically quantifying C losses and accumulation in planted stands. A recent study (*28*) demonstrated that high C stocks do not necessarily reflect high C storage over time, and only relevant approaches such as age-dated cores, unexpectedly uncommon in the literature (*29*), allow measuring C accumulation rates. Rather, the author stresses that “C stock is more of a measure of the amount of C that can be released as CO_2_ and therefore a measure of the vulnerability potential of these C stocks.” Accordingly, the mean C stock buildup ratio *R* of 0.75 indicates considerable C stocks in planted mangrove stands relative to intact stands. In addition, the absence of net loss of soil C stock after planting relative to soil C stocks in intact stands suggests that allochthonous and/or autochthonous soil C sequestration over 40 years of active planting were globally effective in offsetting soil C losses through emission and tidal export reported in bare and recently cleared mangrove areas [e.g., (*30*, *31*)]. Despite this absence of C stock change over time, our model demonstrates that restorable areas may still contain highly significant soil C stocks before planting when compared to their intact counterparts, highlighting the role of conservation of intact mangrove C stocks.

We also found that mixed-species planted stands had higher C stocks than monospecific *Sonneratia*, *Kandelia*, and *Avicennia* spp. stands, a pattern that has also been described for natural mangrove stands (*32*, *33*). However, mean predicted net total biomass C stock gain in *Rhizophora* spp. planted stands was two to three times higher than any other genus and the mixed-species stands, likely due to this genus-specific trait such as dense aerial biomass, deep root system, and relative higher wood specific gravity (*34*). Our results suggest that including *Rhizophora* spp. in mixed-species planted stands would increase C sequestration capacity up to values equal or higher than total biomass C stocks found in intact stands. In contrast, over the first 10 years after planting, species that are fast growing pioneer or resistant to extreme conditions, such as *Sonneratia* and *Avicennia*, presented higher C stock buildup ratios *R* in their BGB relative to *Rhizophora*, *Kandelia*, and mixed-species planted stands. These findings suggest that if planted in the right environmental settings, then these two genera could stabilize soils quickly through ecological facilitation feedbacks to sedimentation while also reducing soil erosion and reducing tidal energy (*35*, *36*).

Here, we deliver guidance for future mangrove restoration and research needs and demonstrate the feasibility to restore up to 75% of mangrove C stocks in 20 to 40 years provided that plantings occur within suitable biophysical conditions (e.g., surface elevation relative to tidal prism and hydroperiod). We also found that fast growing (<10 years to peak biomass) pioneer genera could have important applications for nature-based solutions aiming at soil stabilization, ecological facilitation, and wave energy dampening. While reducing global emissions through industrial decarbonization remains the primary way to reduce GHG emissions and mitigate climate change, conservation of existing C stocks contributes in preventing further emissions. Our models based on 40 years of mangrove forest growth data offer stakeholders insight into the timeline for biomass C stocks to attain levels comparable to intact mangrove stands. Our models also facilitate goal setting; performance measure development; and progress tracking in restoration, rehabilitation, or afforestation projects. Our findings can be useful for nations whose seek in blue economy ways forward to increase adaptive capacity to climate change, meet nationally determined contribution goals, and harness multiple co-benefits inherent to restoration of natural ecological systems.

## MATERIALS AND METHODS

### Experimental design

#### Literature search and exclusion criteria

We systematically reviewed the literature on C stocks in planted (i.e., afforested and restored stands, regardless of the purpose-oriented typology of the planted stands) mangroves to characterize C stock changes over time within the soil (down to 1-m depth), belowground, and aboveground compartments of the ecosystem. To compare the planted sites with intact mangrove ecosystems in the same area, data measured in natural primary stands including intact forest landscapes [PF-IFL, i.e., free of significant human degradation; (*22*)], hereafter called intact, were also recorded.

We collected studies that contained data on C stocks or information allowing us to calculate C stocks in planted mangrove ecosystems [soil bulk density (BD), soil organic C content of soils, biomass, wood density, details on vegetation structure such as tree diameter, basal area, height, density, and species composition]. Those variables were identified from articles or book chapters published in peer-reviewed journals. Published literature search was conducted in Google Scholar, ScienceDirect, and Google using the following keywords: “mangrove + carbon stock + biomass + restored + afforested + plantation.” Additional studies and reports were found by following citations within these published documents. The search resulted in a total of 6850 papers having these keywords.

Published studies or reports that did not present C stock values or information to calculate C stocks and with the following criteria were excluded from the meta-analysis: (i) C stock data published as abstract-only and without any full text that included or referred to a detailed methodology; (ii) data for which the geographic location of the study site could not be found; (iii) stands for which the genera/species were not reported; (iv) stands for which the age of the stand could not be found for planted stands; and (v) data calculated from materials sampled from one single tree/soil core in a study site, without any replication. After applying these exclusion criteria, the number of studies retained for our meta-analysis of C stocks was reduced to 134 studies and reports encompassing a total of 809 planted stands, as well as data from 370 intact stands reported in these studies for comparison. We then conducted a second search to find additional intact stand C stock values nearby the planted stands but which lacked comparison data by country and region using the following keywords “mangrove + carbon stock + biomass + country + region.” After applying for our exclusion criteria, this second search resulted in an additional 52 papers, reports, and theses. Of these, 16 studies reporting on 62 stands were removed from further analyses, having been deemed significantly degraded by the original authors. This left an additional 36 studies on C stocks in intact mangroves and a total of 809 planted stands and 437 intact stands in our final dataset, available within the publicly accessible repository associated with our manuscript. Of these 809 planted stands, 143 were afforested and 666 restored or rehabilitated.

Studies that reported C stock data from recently developed naturally regenerated intact stands were not included in our meta-analysis due to low data availability in the literature (*n* = 74, 49, and 67 for the aboveground, belowground, and soil C stocks in naturally regenerated stands, compared to *n* = 602, 259, and 252 for the aboveground, belowground, and soil C stocks in planted stands). Furthermore, the variability of these data at any given point in time on a global scale was very high, leading to data insensitivity. We arbitrarily included in that category any natural intact primary stands that developed less than 40 years before sampling in the original study, although age is seldom reported or known for mature primary stands (also termed old growth or climax) and not necessarily a suitable indicator of whether a stand has reached a stage of climax or senescence for mangrove forests (*37*).

#### Data collection, handling, and data quality assessment

For each planted mangrove stand, C stocks in each of the soil, sediment, downed wood/ground layer, and AGB and BGB compartments were recorded. Geographic location (latitude and longitude coordinates) of each stand was recorded and converted in decimal degrees. When the geographic coordinates were not stated in the text but the location was described or illustrated as a map, the geographic location of each stand was found using the cloud computing platform Google Earth Engine, and their geographic coordinates were recorded. When the original C stock data were presented as an average across multiple stands within a study site (i.e., a particular delta, estuary, lagoon, open coast, or oceanic island), we recorded the GPS coordinates for the point located in the middle of that study site, i.e., in the middle of the perpendicular line connecting the shoreline to the terrestrial edge of the studied mangrove. The locations of these planted mangrove C stock data (*n* = 809) across mangrove global distribution (*38*) are illustrated in fig. S1.

When available, the age of the stand, tree species composition, and variables used to calculate C stocks were also recovered from each study. Those include the substrate dry BD, organic matter (OM), C content, wood density, tree density, height, and tree basal areas. Data published as illustration only were recovered with Plot Digitizer 2.6.8 software (*39*). We also collected data on the coastal environmental setting of each stand, i.e., delta, oceanic island, estuary, lagoon, and open coast. These were determined on the basis of the geographic coordinates recorded for each study, using the global mangrove biophysical typology developed in (*40*) applied to maps of global mangrove extent generated by Global Mangrove Watch (*38*). Each data entry was further categorized into coastal environmental setting and the specific diversity of each planted mangrove stands (i.e., genus and species name or mixed-species planted stands).

### C stock data quality assessment and C stock data calculation

Major effort was made to standardize C stock data measurements in each compartment of the mangrove ecosystem (*41*). However, differences in methodology in most publications before 2012 and the need to avoid destructive methods in restored/afforested stands leave little chance for an easy comparison between mangrove stands and may introduce significant bias in meta-analyses. We identified the measurement bias that could affect the C stock data quality of each collected study and thus the reliability and validity of our meta-analysis based on the following criteria [adapted from (*33*)]: the type of plant material sampled; the core depth for soil C stock data; the extent of the area sampled; the method to calculate C content (chemical analysis or function of the biomass or OM); and the type of allometric equation used to calculate C stocks. These criteria, discussed below, can be consulted in tables S3 to S5, along with the corresponding % of data that they represent in the final dataset.

Among the planted stands, we recorded twice as much C stock data for the aboveground compartment than for either the belowground or the soil compartments (see Fig. 1). Data availability on downed wood C in mangrove planted stands found in the literature was too low (*n* = 28) to include this component in our dataset, and the aboveground C stocks should therefore be regarded as conservative.

Of the soil C stock data meeting our inclusion criteria, 40% of the data were measured/calculated along a profile of 1-m depth, 36% along a profile < 1 m and 24% along a profile > 1-m depth (maximum depth value of 4 m). We scaled all C stock data to 1-m depth by extrapolating or interpolating these data to 1 m (although most studies that collected soil cores down to a profile > 1-m depth also reported C stocks at 1 m and therefore did not need scaling). We tested two scaling methods: (i) by multiplying the average C content (in %) by the dry BD (in grams per cubic centimeter) and then adjusting that value to 1 m [as in (*33*)] and (ii) by dividing the known C stock by the sampled depth and then multiplying it to 1 m. We compared both methods using soil core data that included observed C stock values to 1-m depth as well as at shallower or deeper depths and for which dry BD was reported (*n* = 169). We then regressed these observed values versus the predicted values compiled with both approaches and compared the *R*^2^ and slopes of the regressions (see fig. S2). The coefficient of determination and the slope for the second scaling approach based on soil depth only were both closer to 1 (*R*^2^ = 0.60, slope = 0.74, best fitted trend linear) compared to the first scaling approach based on BD and C content (*R*^2^ = 0.38, slope = not significant, best fitted trend polynomial). Therefore, we used the second scaling approach to extrapolate our data to 1-m depth. It is worth noting that a recent study encompassing large climatic and biogeophysical gradients reported that mangrove soil depth ranges from 22 to 300 cm, with mean value of 216 cm, and that only 13% of mangrove soils have a mean soil depth of ≤1 m (*7*). According to that study, it is therefore highly likely that global soil C stock values from mangroves represent underestimates by up to 50%.

When the soil C stock data were not reported in a study, we calculated soil C stocks by multiplying the dry BD by the soil depth interval and by the C content (*41*). If the BD and C content were reported for several depth intervals of a same core, then we calculated the C stock for each interval and then summed the C mass of each of the sampled soil depths.

About 83% of the soil C stock data used in our study were calculated using C content determined via chemical extraction (table S5), whereas 17% of the original data were presented as a function of OM content (measured by loss of ignition), introducing possible bias the original data (*41*). In contrast, few of the original AGB or BGB C stock data in our dataset were calculated on the basis of actual C content analysis of the plant tissues. Instead, 57% of the belowground and 90% of the aboveground C stock data relied on C content calculated as a general function of the biomass published in the literature (*41*–*43*), sometimes indiscriminately between BGB and AGB and without distinction between plant materials (tables S3 and S4). When C content specific to the targeted region/local area or for a specific species was found in another study, we recalculated the corresponding C stocks.

Uncertainties in the original studies also included the use of general allometric equations (nonspecific to the targeted study site or to a species) to calculate the BGB and AGB. This was the case for 19.6% of our belowground C stock data and for 18.5% of our aboveground C stock data (see tables S3 and S4). Similarly, ~60% of the belowground and aboveground C stock data were calculated on the basis of published general allometric equations developed for a particular mangrove genus or species. As the structural characteristics of a particular mangrove species may vary significantly between and even within study locations, the use of general allometric equations can lead to inaccuracies (*41*). Therefore, if a general allometric equations was used in the original study and if a specific allometric equation was found in another publication for that same study site or in a nearby area and for the same species, then we recalculated the corresponding biomass based on this more specific allometric equation.

Seventy-eight percent of the BGB estimates in our dataset rely on general allometric equations related to AGB-related attributes (e.g., tree density, height, diameter at breast height, [DBH], and wood density). A review comparing mangrove BGB values from direct field measurements and estimations from allometric equations reported that this systematic bias could introduce an uncertainty of 4 to 15% in the total ecosystem C stock (*44*). Where possible, we recalculated the BGB on the basis of the root-to-shoot relationship established by (*44*). However, this was possible only for 10% of the belowground dataset.

Last, only a minor percentage of the data collected for the BGB, AGB, and soil C stocks were collected according to a sampling design aiming to maximize the exploration of an entire planted stand/study site (tables S3 to S5). This implies that C stock variations driven by local biogeochemical factors related to the environmental gradient characteristic of many mangrove areas (e.g., tidal pumping, elevation-related characteristics such as salinity, OM decomposition rate, and nutrient content) were not necessarily captured by the C stock ratios calculated in our models.

#### C stock ratio calculation

To assess how effective mangrove planting efforts are at returning C stocks to those of intact stands in the same geographic location, a meta-analysis was performed on the C stock buildup ratio, *R*, of planted to intact mangrove stands for each of the aboveground, belowground, and soil compartments. In addition, we choose to compare C stock of planted stands only with those of intact stands developing in the same geomorphological class, stratified into estuary, delta, lagoon, open coast, or oceanic island (regardless of the specific composition of the intact stand). Geomorphology has been shown to influence the load of allochthonous materials, burial rates, and soil C sequestration in mangrove ecosystems [e.g., (*6*, *45*, *46*)]. Geomorphology is also a key factor in the availability of essential nutrients, especially nitrogen and phosphorous, which, in turn, influences the allocation of biomass to belowground versus aboveground tree structures in mangrove species (*6*, *47*–*49*). This approach reduces bias linked with climatic or geomorphological characteristics that may occur at different spatial scales.

We first calculated the C stock buildup ratios relative to intact mangrove stands for each planted mangrove stand within the same location and the same geomorphological class (maximum distance threshold of ~10 km for the largest sites). When several intact mangrove plots were recorded in the same location, the C stocks of a planted mangrove stand were divided by the mean C stock of all the available intact mangrove plots. For mangrove stands planted away from any intact mangrove forest or for which there was no intact C stock data available for a particular compartment of the ecosystem, C stock ratios were calculated relative to the closest intact mangrove stands that belong to the same climatic and geomorphological class of the targeted mangrove stands. However, this only applied to 12 of the 181 locations. The mean distance value of these selected remote locations in our dataset is 313 km (range, 23 to 385 km). For 12 locations (encompassing 124 stands), we did not find C stock data collected in intact stands nearby or in remote areas that belonged to the same climatic and geomorphological class than that of the targeted mangrove stands. These stands were therefore removed from further analyses. In addition, too few data were found on soil C stock ratios for *Avicennia* planted stands (*n* = 27 reported by five studies). Therefore, soil C stock data for *Avicennia* were not analyzed further. This left a total of 684 planted stands used in our final logistic C stock buildup ratio models [AGB C stock buildup ratios (*n* = 602), BGB C stock buildup ratios (*n* = 259), and soil C stock buildup ratios (*n* = 252)]. Of these 684 planted stands, 121 were afforested and 563 were restored or rehabilitated. The details of the intact mangrove stands used to calculate the C stock buildup ratio in each planted stand (geographic location, distance from the planted stand, coastal setting, diversity, and C stocks) are available within the publicly accessible repository associated with our manuscript.

One limitation of our approach is that we did not compare C stock data of planted mangrove stands with that of intact stands located in a similar position along the intertidal, riverine, or elevation gradient in the same geographic location. While elevation and position along riverine and intertidal gradient have been reported to influence physicochemical factors that play a role in species zonation, productivity, and soil C sequestration in many studies [e.g., (*27*, *46*, *50*)], pairing planted stands with intact stands based on these factors was not universally possible. Often, all these attributes were not present, and, if available, attributes were not necessarily recorded on the same site or even in the same geographic location. Moreover, while determining position along the intertidal gradient may be straightforward in open coast areas, distinction is more complex in deltas and estuaries where multiple water courses (creeks and rivers) can be found, and soil physicochemical properties that control C stocks are too variable to standardize (*50*). Because delta and estuarine planted stands constitute the largest part of our dataset (*n* delta = 408, *n* estuary = 235, *n* lagoon = 23, and *n* open coast = 139), this could have inserted a bias in our analyses.

### Statistical analysis

#### Logistic models for C stock ratios buildup rates

We sought a model for C stock buildup in afforested and restored mangrove stands (*n* = 684 stands) based on the planted to intact C stock ratios, *R*, described in the previous section. The initial idea being that there is an average C stock value for intact mangrove forests in a given location and that, over time, C stock of planted stands in the vicinity would reach to values similar to those of these intact stands (*R* = 1). This leads to a logistic model for C stock buildup. However, upon graphical inspection of the collected data, C stocks in some planted stands do not appear to reach to this average C stock value, while other planted stands build up C stock much larger than at the intact stands. Therefore, we wanted our model to allow for the possibility that, under some conditions, planted mangrove stands build up C stocks that are lower or higher than that of the intact stands. This led to the following logistic model (Eq. 1)

$$C_{d,\mathrm{ij}}=\frac{C_{u,\mathrm{ij}}R{\text{max}}_{j}}{1+\text{exp}\left[-\beta_{0j}-\beta_{1j}\text{log}\left({\text{age}}_{\mathrm{ij}}\right)\right]}$$ (1)

where *i* indexes site, *j* indexes site type, *C*_*d*, *ij*_ is the C stock for the planted site, *C*_*u*, *ij*_ is the C stock for the intact site, age*_ij_* is the age of the planted site, *R*max*_j_* is the C stock ratio of planted to intact sites for infinite age*_ij_*, β_1*j*_ determines the slope of the logistic curve, and β_0*j*_ determines the location of the logistic curve.

From this logistic curve, we can build the following statistical model (Eq. 2)

$$\begin{array}{c} \text{log}\frac{C_{d,\mathrm{ij}}}{C_{u,\mathrm{ij}}}∼\text{Normal}(\text{log}\left(R{\text{max}}_{j}\right)-\\ \text{log}\left\{1+\text{exp}\left[-\beta_{0j}-\beta_{1j}\text{log}\left({\text{age}}_{\mathrm{ij}}\right)\right]\right\},\sigma_{j}) \end{array}$$ (2)

where σ*_j_* is the error SD. We implemented this model in a Bayesian generalized nonlinear modeling framework. Because we took a Bayesian approach, we choose priors to incorporate the degree of (un)certainty surrounding the model parameters. We chose them to be log*R*max*_j_* ~ Normal(0,2.5), β_0*j*_ ~ Normal(0,5), β_1*j*_ ~ Normal(0,2.5), and logσ*_j_* ∝ 1.

The priors on β_0*j*_ and β_1*j*_ were chosen because their hyperparameter values have been shown to work well in logistic curve models (*51*). However, we chose a normal distribution instead of a Cauchy distribution because the tails are not as heavy. Specifically, the prior on β_0*j*_ could be thought of as the prior on the log odds when log age = 0, i.e., when age = 1. Ninety-five percent of the prior probability of this distribution is between −10 and 10 for the log odds or between 0.0001 and 0.9999 on the logistic curve. The prior on β_1*j*_ places 95% of the prior probability so that a unit change in the log age will result in a change in the log-odds ratio between −5 and 5. In other words, we expect the size of the recovery or buildup to be at most from 50 to 99% after 2 to 3 years (specifically *e* years). These logistic priors also work well on log means (*51*), such as log *R*max. It places 95% of the prior probability on values of *R* between 0.001 and 148, which in retrospect might have been too diffuse. However, it places about 70% of the prior probability on values of *R* between 0.08 and 12, which is a reasonable range considering the maximum *R* observed was 3.84. Last, the prior for logσ*_j_* was chosen because it is the Jefferey’s prior and the default within the brms package provided.

Because we took a Bayesian approach, we can use samples from the posterior distribution of the parameters to compute the posterior distribution of the age when a site of a particular type reaches a particular ratio of planted to intact mangrove stand C stock. For this, we used the following equation (Eq. 3)

$$\text{age}=\text{exp}\left[\frac{\text{log}\left(\frac{R}{R{\text{max}}_{j}-R}\right)-\beta_{0,j}}{\beta_{1,j}}\right]$$ (3)

where *R* is a C stock ratio of planted to intact sites for which the age is being computed. Note that this equation is undefined when $R{\text{max}}_{j}\leq R$ but could be treated as having infinite age for these values.

The Bayesian generalized nonlinear model was implemented using the brm function in the brms version 2.16.3 package (*52*, *53*) in R version 4.1.1 computer programming language (*54*). For each model, the specific value of each prior (on β_0*j*_, β_1*j*_, and logσ*_j_*) can be consulted in table S6.

## Supplementary Materials

### This PDF file includes:

Figs. S1 and S2 Tables S2 to S6 Legend for table S1 Legend for data S1 References
