# Supplementary material for: Four decades of data indicate that planted mangroves stored up to 75% of the carbon stocks found in intact mature stands
Source: Sci Adv. 2024 Jul 5;10(27):eadk5430. doi: 10.1126/sciadv.adk5430 (PMC11801255; doi:10.1126/sciadv.adk5430)
Supplement: Supplementary file 1 — Figs. S1 and S2 Tables S2 to S6 Legend for table S1 Legend for data S1 References [file sciadv.adk5430_sm.pdf]

Science Advances
AAAS

Supplementary Materials for

Four decades of data indicate that planted mangroves stored up to 75% of the carbon stocks found in intact mature stands

Carine F. Bourgeois *et al.*

Corresponding author: Richard A. MacKenzie, richard.mackenzie@usda.gov

Sci. Adv. **10**, eadk5430 (2024)
DOI: 10.1126/sciadv.adk5430

The PDF file includes:

Figs. S1 and S2
Tables S2 to S6
Legend for table S1
Legend for data S1
References

Other Supplementary Material for this manuscript includes the following:

Table S1
Data S1

fig. S1.

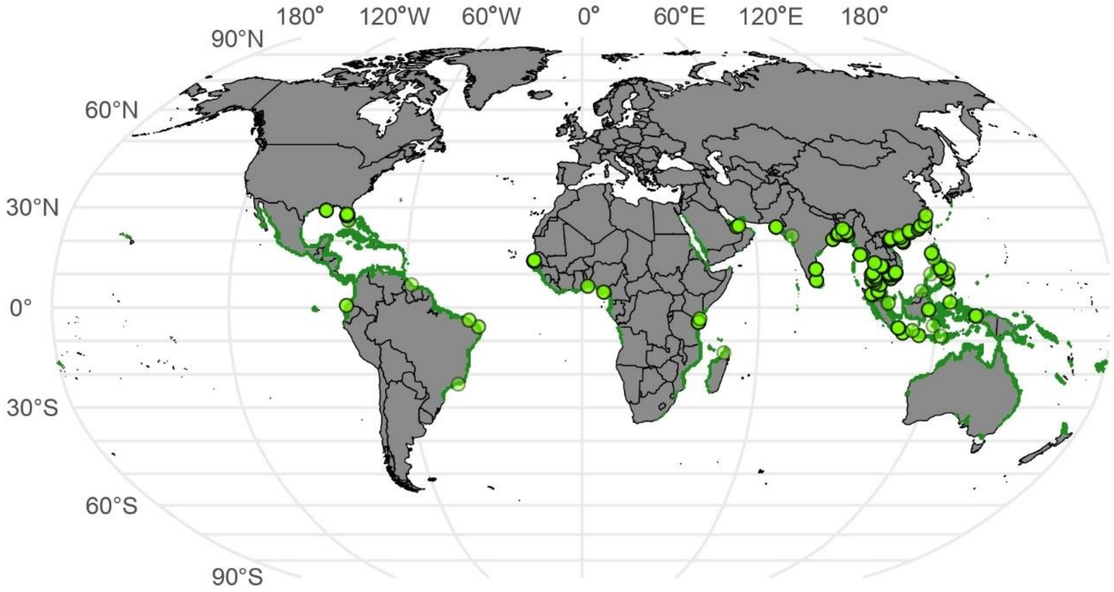

World map indicating the locations of planted mangrove C stock data (Mg ha^{-1}) (light green circles, n = 809). A darker shade of light green indicates areas with a higher number of observations. The dark green outline along continents' edges indicates mangrove global distribution (*38*)

fig. S2.

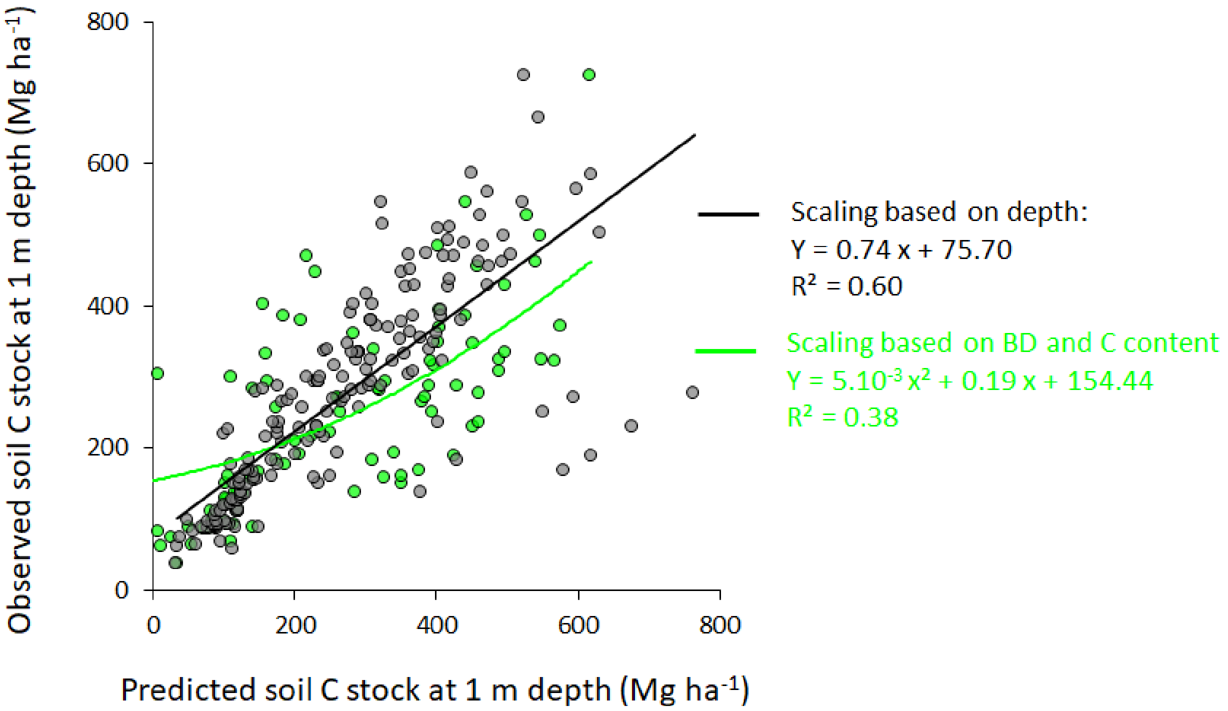

Scatter plots illustrating the comparison between two scaling methods to predict soil C stock data down to 1 m depth. Both models were compiled using data from soil cores sampled to both 1 m depth and to shallower or larger depths. The green circles indicate predicted C stock values at 1 m depth (X axis) – calculated by multiplying the mean bulk density (BD) and C content from known depths (≠ 1 m) and then multiplying it to 1 m depth – plotted against known C stock from the same cores at 1 m depth (i.e. observed values, Y axis). The black circles indicate the predicted C stock values at 1 m depth – calculated by dividing the C stock values by the depth of the samples (≠ 1 m) and then multiplying it to 1 m – plotted against known C stock from the same cores at 1 m depth (i.e. observed values, Y axis). The polynomial trend (green curve) and linear trend (black line) represent the best fitted polynomial and linear trend for each model, respectively. For each regression analysis, the equation and R^2 are indicated

table S2.

Maximum planted:intact C stocks ratios R_{max} (95% C.I.) compiled from the logistic growth models for all planted mangrove stands, and then for mixed-species planted stands and for the most common genera used for monospecific planted stands. For each model, the age at R_{max} is indicated, along the mean (and 95% C.I.) global predicted net carbon stock gain (MgC ha^{-1}) over a 40 years period of time

	Soil (non-significant)			AGB			BGB		
	R_{max} (95% C.I)	Age at R_{max} (years)	Net C gain (Mg ha^{-1}) (95% C.I)	R_{max} (95% C.I)	Age at R_{max} (years)	Net C gain (Mg ha^{-1}) (95% C.I)	R_{max} (95% C.I)	age at R_{max} (years)	Net C gain (Mg ha^{-1}) (95% C.I)
All data	0.75 (0.67–0.82)	40	74.08 (27.69–94.06)	0.71 (0.57–0.91)	40	44.52 (35.85–57.20)	0.73 (0.58–0.94)	40	24.61 (19.49–31.60)
Mixed-species	0.91 (0.80–1.03)	8.5	6.09 (-3.30–29.39)	0.92 (0.45–1.81)	40	57.41 (28.27–113.53)	0.67 (0.48–1.03)	40	22.56 (16.14–34.49)
Rhizophora spp.	0.96 (0.86–1.07)	11.7	12.32 (1.02–82.46)	1.62 (1.20–2.28)	40	101.60 (75.65–143.29)	1.62 (0.56–4.23)	40	54.56 (18.82–141.99)
Sonneratia spp.	0.66 (0.49–0.90)	27.2	24.10 (17.04–119.94)	0.67 (0.42–1.50)	40	41.07 (26.41–90.53)	0.72 (0.51–1.62)	40	24.20 (17.15–51.84)
Kandelia spp.	0.64 (0.50–1.00)	40	74.65 (69.03–80.47)	0.44 (0.29–0.79)	40	27.74 (18.24–49.59)	0.56 (0.25–1.40)	40	18.15 (8.40–44.78)
Avicennia spp.	NA	NA	NA	0.39 (0.25–0.90)	40	24.54 (15.84–56.59)	0.80 (0.54-1.59)	40	26.83 (18.16–53.01)

table S3.

Overview of the methodology quality used by the authors of the collected studies to measure and calculate carbon (C) stocks in the aboveground compartment in the planted mangrove stand dataset, presented here as the number of stands collected prior the application of any exclusion criteria of these stands in our final analysis (n) and as the % of the entire dataset for each criteria. Criteria for C stocks measurements in the aboveground biomass include whether allometric equations are specific to the species and location sampled; the type of biological material sampled; the means used to calculate C within the biomass and the extent and representativeness of the area sampled

**ABOVEGROUD C STOCK**		
**Allometric equations used to calculate biomass**	**n**	**%**
biomass data calculated based on allometric equation developed for that particular planted stand/study site	269	20.4
biomass data calculated based on published general allometric equation developed for a particular genera/species	805	61.1
biomass data calculated based on published general allometric equation for mangroves (no specific species)	244	18.5
n total	1318	100.0
**material sampled**	**n**	**%**
C stock data calculated for dead and live aboveground pools, including: trees - palms - shrubs/dwarf mangroves; seedlings (< 1.37 m height) /herbs/litter/pneumatophores; downed wood	374	28.4
C stock data calculated for living and dead standing trees/palms/ferns only	297	22.5
C stock data calculated for living standing trees/palms/ferns only	647	49.1
n total	1318	100.0
**C analysis**	**n**	**%**
Original data presented as C density, stocks, or % OC measured through chemical extraction	75	5.7
C data calculated as a function of biomass, with distinction made between different plant materials (leaves, branches, stems, aerial roots)	69	5.2
C data calculated as a function of biomass, without distinction between different plant materials (leaves, branches, stems, aerial roots)	1174	89.1
n total	1318	100.0
**area sampled**	**n**	**%**
C stock data given or calculable for several individual plots as part of a sampling design that maximize the exploration of the entire planted stand/study site (e.g. multiple areas along an intertidal or estuarine gradient)	240	18.2
C stock data given or calculable for several individual plots in the planted stand /study site	1030	78.1
C stock data presented as an average of multiple plots in the planted stand /study site	48	3.6
C stock data measured for one single plot and then extrapolated to the entire planted stand /study site	0	0.0
n total	1318	100.0

table S4.

Overview of the methodology quality used to measure and calculate carbon (C) stocks for the belowground compartment in the planted mangrove stand dataset, presented here as the number of stands collected prior the application of any exclusion criteria of these stands in our final analysis (n) and as the % of the entire dataset for each criteria. Criteria for C stocks measurements in the belowground biomass include whether allometric equations are specific to the species and location sampled; the type of biological material sampled; the means used to calculate C within the biomass and the extent and representativeness of the area sampled

**BELOWGROUND C STOCK**		
**Allometric equations used to calculate biomass**	**n**	**%**
biomass data calculated based on allometric equation developed for that particular planted stand/study site	164	22.0
biomass data calculated based on published general allometric equation developed for a particular genera/species	435	58.4
biomass data calculated based on published general allometric equation for mangroves (no specific species)	146	19.6
n total	745	100.0
**C analysis**	**n**	**%**
Original data presented as C density, stocks, or % OC measured through chemical extraction	44	5.9
C data calculated as a function of biomass specific to roots	274	36.8
C data calculated as a function of biomass, without distinction between different plant materials (roots, leaves, branches, stems, aerial roots)	427	57.3
n total	745	100.0
**area sampled**	**n**	**%**
C stock data given or calculable for several individual plots as part of a sampling design that maximize the exploration of the entire planted stand /study site (e.g. multiple areas along an intertidal or estuarine gradient)	119	16.0
C stock data given or calculable for several individual plots in the planted stand /study site	580	77.9
C stock data presented as an average of multiple plots in the planted stand/study site	46	6.2
C stock data measured for one single plot and then extrapolated to the entire planted stand/study site	0	0.0
n total	745	100.0

table S5.

Overview of the methodology quality used to measure and calculate carbon (C) stocks for the soil compartment in the planted mangrove stand dataset, presented here as the number of stands collected prior the application of any exclusion criteria of these stands in our final analysis (n) and as the % of the entire dataset for each criteria. Criteria for C stocks measurements in soil include the number of soil cores sampled, their depth and the means used to analyze or calculate the C content and the bulk density, as well as the extent and representativeness of the area sampled

**SOIL C STOCK**		
**downcore soil depth**	**n**	**%**
C stock given or calculable down to 80 - 100 cm depth	532	71.2
C stocks given or calculable to 41 - 79 cm depth and then extrapolated	102	13.7
stocks given or calculable for the surface (< 40 cm) and then extrapolated	113	15.1
n total	747	100.0
**Quality of percent organic C data**	**n**	**%**
Original data presented as C density, stocks, or % OC measured through chemical extraction	619	82.9
Original data presented as a function of organic matter (OM) content	128	17.1
Original data presented as total carbon	0	0.0
Global average used	0	0.0
n total	747	100.0
**bulk density**	**n**	**%**
BD given to 100 cm	451	60.4
BD given to < 100 cm depth and then extrapolated	264	35.3
BD estimated from a pedotransfer function	32	4.3
n total	747	100.0
**area sampled**	**n**	**%**
C stock data given or calculable for several individual plots as part of a sampling design that maximize the exploration of the entire planted stand/study site (e.g. multiple areas along an intertidal or estuarine gradient)	227	30.4
C stock data given or calculable for several individual plots in the planted stand/study site	450	60.2
C stock data presented as an average of multiple plots in the planted stand/study site	70	9.4
C stock data measured for one single plot and then extrapolated to the entire planted stand/study site	0	0.0
n total	747	100.0

table S6.

Values of each estimate (R_{max}, priors on $\beta_{0\,j}$, $\beta_{1\,j}$ and σ_j) of the logistic growth models of the C stock recovery ratios over time in the aboveground biomass, belowground biomass and soil for the entire data set and for the different genera and mixed-species mangrove planted stands relative to intact mangrove stands

model	C stock	R_{max}	β_0	β_1	σ	figure reference
all data	aboveground	0.728	-6.384	2.777	1.402	Fig. 2 A
all data	belowground	0.740	-6.430	3.148	1.165	Fig. 2 A
all data	soil	0.748	3.104	3.059	0.748	Fig. 2 A
mixed-species	aboveground	2.551	-6.038	1.585	1.625	Fig. 3 A
mixed-species	belowground	0.689	-6.496	3.051	0.795	Fig. 3 A
mixed-species	soil	0.966	5.606	1.010	0.479	Fig. 3 A
Avicennia spp.	aboveground	0.457	-5.867	3.249	1.248	Fig. 3 B
Avicennia spp.	belowground	0.875	-6.573	3.641	0.939	Fig. 3 B
Avicennia spp.	soil	NA	NA	NA	NA	Fig. 3 B
Kandelia spp.	aboveground	0.455	-5.809	3.865	1.088	Fig. 3 C
Kandelia spp.	belowground	1.027	-4.243	1.655	1.022	Fig. 3 C
Kandelia spp.	soil	0.824	1.750	1.597	0.673	Fig. 3 C
Rhizophora spp.	aboveground	2.101	-6.409	2.104	0.877	Fig. 3 D
Rhizophora spp.	belowground	6.275	-6.915	1.662	1.584	Fig. 3 D
Rhizophora spp.	soil	1.003	5.049	1.292	0.315	Fig. 3 D
Sonneratia spp.	aboveground	1.029	-3.696	1.932	1.064	Fig. 3 E
Sonneratia spp.	belowground	0.995	-3.301	2.444	0.816	Fig. 3 E
Sonneratia spp.	soil	0.756	3.738	1.390	0.766	Fig. 3 E

Supplemental auxiliary files

table S1 (separate xlsx file)
Statistical summary of the aboveground (AGB), belowground (BGB) and soil carbon (C) stocks across age classes (in years) of PF-IFL stands (primary stands including intact forest landscape, comprising “mature” intact and naturally regenerated stands) and planted mangrove stands collected in the database. C stock data are indicated in MgC ha^{-1}. For each category, the number of C stock values in the collected stands (n) are also indicated, followed by the mean, standard deviation, and minimum and maximum C stock values

Data S1. (separate xlsx file)

Belowground biomass and belowground biomass C stock data compiled by Rovai et al. (*55*), used in the present study as global mean belowground biomass C stock in intact mangroves and to assess predicted global C stock gain in mangrove planted stands from our models. The authors used a biomass to carbon stock conversion factor of 0.475 (as in *42*). For each data entry, the name and geographic coordinates of the location are given, along with the biomass within the coarse (> 2mm) and fine (< 2mm) dead and live roots, and the total C stock

REFERENCES AND NOTES

1. L. Goldberg, D. Lagomasino, N. Thomas, T. Fatoyinbo, Global declines in human-driven mangrove loss. *Glob. Chang. Biol.* **26**, 5844–5855 (2020).

2. N. C. Duke, J. O. Meynecke, S. Dittmann, A. M. Ellison, K. Anger, U. Berger, S. Cannicii, K. Diele, K. C. Ewel, C. D. Field, N. Koedam, S. Y. Lee, C. Marchand, I. Nordhaus, F. Dahdouh-Guebas, A world without mangroves?. *Science* **317**, 41–42 (2007).

3. N. Thomas, R. Lucas, P. Bunting, A. Hardy, A. Rosenqvist, M. Simard, Distribution and drivers of global mangrove forest change, 1996–2010. *PLOS ONE* **12**, e0179302 (2017).

4. S. E. Hamilton, D. Casey, Creation of a high spatio-temporal resolution global database of continuous mangrove forest cover for the 21st century (CGMFC-21). *Glob. Ecol. Biogeogr.* **25**, 729–738 (2016).

5. A. Komiyama, J. E. Ong, S. Poungparn, Allometry, biomass, and productivity of mangrove forests: A review. *Aquat. Bot.* **89**, 128–137 (2008).

6. R. R. Twilley, A. S. Rovai, P. Riul, Coastal morphology explains global blue carbon distributions. *Front. Ecol. Environ.* **16**, 503–508 (2018).

7. J. B. Kauffman, M. F. Adame, V. B. Arifanti, L. M. Schile-Beers, A. F. Bernardino, R. K. Bhomia, D. C. Donato, I. C. Feller, T. O. Ferreira, M. del Carmen Jesus Garcia, R. A. MacKenzie, J. P. Megonigal, D. Murdiyarso, L. Simpson, H. H. Trejo, Total ecosystem carbon stocks of mangroves across broad global environmental and physical gradients. *Ecol. Monogr.* **90**, e01405 (2020).

8. R. J. Temmink, L. P. Lamers, C. Angelini, T. J. Bouma, C. Fritz, J. van de Koppel, R. Lexmond, M. Rietkerk, B. R. Silliman, H. Joosten, T. van der Heide, Recovering wetland biogeomorphic feedbacks to restore the world’s biotic carbon hotspots. *Science* **376**, eabn1479 (2022).

9. S. Song, Y. Ding, W. Li, Y. Meng, J. Zhou, R. Gou, C. Zhang, S. Ye, N. Saintilan, K. W. Krauss, S. Crooks, S. Lv, G. Lin, Mangrove reforestation provides greater blue carbon benefit than afforestation for mitigating global climate change. *Nat. Commun.* **14**, 756 (2023).

10. E. Bayraktarov, M. I. Saunders, S. Abdullah, M. Mills, J. Beher, H. P. Possingham, P. J. Mumby, C. E. Lovelock, The cost and feasibility of marine coastal restoration. *Ecol. Appl.* **26**, 1055–1074 (2016).

11. R. R. Lewis III, B. M. Brown, L. L. Flynn, "Methods and criteria for successful mangrove forest rehabilitation" in *Coastal Wetlands* (Elsevier, 2019), pp. 863–887.

12. M. J. Osland, A. C. Spivak, J. A. Nestlerode, J. M. Lessmann, A. E. Almario, P. T. Heitmuller, M. J. Russel, K. W. Krauss, F. Alvarez, D. D. Dantin, J. E. Harvey, A. S. From, N. Cormier, C. L. Stagg, Ecosystem development after mangrove wetland creation: Plant–soil change across a 20-year chronosequence. *Ecosystems* **15**, 848–866 (2012).

13. M. J. Osland, L. C. Feher, A. C. Spivak, J. A. Nestlerode, A. E. Almario, N. Cormier, A. S. From, K. W. Krauss, M. J. Russel, F. Alvarez, D. D. Dantin, J. E. Harvey, C. L. Stagg, Rapid peat development beneath created, maturing mangrove forests: Ecosystem changes across a 25-yr chronosequence. *Ecol. Appl.* **30**, e02085 (2020).

14. A. G. DelVecchia, J. F. Bruno, L. Benninger, M. Alperin, O. Banerjee, J. de Dios Morales, Organic carbon inventories in natural and restored Ecuadorian mangrove forests. *PeerJ* **2**, e388 (2014).

15. K. W. Krauss, N. Cormier, M. J. Osland, M. L. Kirwan, C. L. Stagg, J. A. Nestlerode, M. J. Russel, A. S. From, A. C. Spivak, D. D. Dantin, J. E. Harvey, A. E. Almario, Created mangrove wetlands store belowground carbon and surface elevation change enables them to adjust to sea-level rise. *Sci. Rep.* **7**, 1–11 (2017).

16. M. F. Adame, R. M. Zakaria, B. Fry, V. C. Chong, Y. H. A. Then, C. J. Brown, S. Y. Lee, Loss and recovery of carbon and nitrogen after mangrove clearing. *Ocean Coast. Manag.* **161**, 117–126 (2018).

17. S. Sharma, R. A. MacKenzie, T. Tieng, K. Soben, N. Tulyasuwan, A. Resanond, G. Blate, C. M. Litton, The impacts of degradation, deforestation and restoration on mangrove ecosystem carbon stocks across Cambodia. *Sci. Total Environ.* **706**, 135416 (2020).

18. D. Murdiyarso, S. D. Sasmito, M. Sillanpää, R. A. MacKenzie, D. Gaveau, Mangrove selective logging sustains biomass carbon recovery, soil carbon, and sediment. *Sci. Rep.* **11**, 1–10 (2021).

19. J. Boone Kauffman, V. B. Arifanti, H. Hernandez Trejo, M. del Carmen Jesús García, J. Norfolk, M. Cifuentes, D. Hadriyanto, D. Murdiyarso, The jumbo carbon footprint of a shrimp: Carbon losses from mangrove deforestation. *Front. Ecol. Environ.* **15**, 183–188 (2017).

20. United Nations, New UN Decade on Ecosystem Restoration (2020); www.decadeonrestoration.org/.

21. T. Worthington, M. Spalding, Mangrove restoration potential: A global map highlighting a critical opportunity (2018), Apollo - University of Cambridge Repository (2018); https://doi.org/10.17863/CAM.39153.

22. International Union for Conservation of Nature, IUCN Policy Statement on Primary Forests (2020); https://www.iucn.org/sites/default/files/2022-05/iucn-policy-statement-for-primary-forests.pdf

23. UK Department for Transport, Vehicle licensing statistics data files, UK government repository (2022); www.gov.uk/government/statistics/vehicle-licensing-statistics-july-to-september-2022.

24. H. Ritchie, M. Roser, P. Rosado, CO_2 and Greenhouse Gas Emissions, Our World In Data (2020); https://ourworldindata.org/co2-and-greenhouse-gas-emissions.

25. R. H. Suello, S. L. Hernandez, S. Bouillon, J. P. Belliard, L. Dominguez-Granda, M. Van de Broek, A. M. Rosado Moncayo, J. R. Veliz, K. P. Ramirez, G. Govers, S. Temmerman, Mangrove sediment organic carbon storage and sources in relation to forest age and position along a deltaic salinity gradient. *Biogeosciences* **19**, 1571–1585 (2022).

26. J. O. Bosire, F. Dahdouh-Guebas, M. Walton, B. I. Crona, R. R. Lewis III, C. Field, J. G. Kairo, N. Koedam, Functionality of restored mangroves: A review. *Aquat. Bot.* **89**, 251–259 (2008).

27. A. Lunstrum, L. Chen, Soil carbon stocks and accumulation in young mangrove forests. *Soil Biol. Biochem.* **75**, 223–232 (2014).

28. T. C. Jennerjahn, Relevance and magnitude of 'Blue Carbon' storage in mangrove sediments: Carbon accumulation rates vs. stocks, sources vs. sinks. *Estuar. Coast. Shelf Sci.* **247**, 107027 (2020).

29. S. Sharma, R. Ray, C. Martius, D. Murdiyarso, Carbon stocks and fluxes in Asia-Pacific mangroves: Current knowledge and gaps. *Environ. Res. Lett.* **18**, 044002 (2023).

30. S. D. Sasmito, P. Taillardat, J. N. Clendenning, C. Cameron, D. A. Friess, D. Murdiyarso, L. B. Hutley, Effect of land-use and land-cover change on mangrove blue carbon: A systematic review. *Glob. Chang. Biol.* **25**, 4291–4302 (2019).

31. L. C. Z. Jimenez, H. M. Queiroz, X. L. Otero, G. N. Nóbrega, T. O. Ferreira, Soil organic matter responses to mangrove restoration: A replanting experience in Northeast Brazil. *Int. J. Environ. Res. Public Health* **18**, 8981 (2021).

32. R. A. MacKenzie, P. B. Foulk, J. V. Klump, K. Weckerly, J. Purbospito, D. Murdiyarso, D. C. Donato, V. N. Nam, Sedimentation and belowground carbon accumulation rates in mangrove forests that differ in diversity and land use: A tale of two mangroves. *Wetl. Ecol. Manag.* **24**, 245–261 (2016).

33. T. B. Atwood, R. M. Connolly, H. Almahasheer, P. E. Carnell, C. M. Duarte, C. J. Ewers Lewis, X. Irigoien, J. J. Kelleway, P. S. Lavery, P. I. Macreadie, O. Serrano, C. J. Sanders, I. Santos, A. D. L. Steven, C. E. Lovelock, Global patterns in mangrove soil carbon stocks and losses *Clim. Change* **7**, 523–528 (2017).

34. A. E. Zanne, G. Lopez-Gonzalez, D. A. Coomes, J. Ilic, S. Jansen, S. L. Lewis, R. B. Miller, N. G. Swenson, M. C. Wiemann, J. Chave, Towards a worldwide wood economics spectrum dataset, Driad Repository (2009); https://doi.org/10.5061/dryad.234.

35. M. P. Kumara, L. P. Jayatissa, K. W. Krauss, D. H. Phillips, M. Huxham, High mangrove density enhances surface accretion, surface elevation change, and tree survival in coastal areas susceptible to sea-level rise. *Oecologia* **164**, 545–553 (2010).

36. J. K. S. Langat, B. K. Kirui, M. W. Skov, J. G. Kairo, M. Mencuccini, M. Huxham, Species mixing boosts root yield in mangrove trees. *Oecologia* **172**, 271–278 (2013).

37. A. E. Lugo, Old-growth mangrove forests in the United States. *Conserv. Biol.* **11**, 11–20 (1997).

38. P. Bunting, A. Rosenqvist, R. M. Lucas, L. M. Rebelo, L. Hilarides, N. Thomas, A. Hardy, T. Itoh, M. Shimada, C. M. Finlayson, The global mangrove watch–a new 2010 global baseline of mangrove extent. *Remote Sens.* **10**, 1669 (2018).

39. J. A. Huwaldt, S. Steinhorst, Plot Digitizer, version 2.6.8, Sourceforge (2015); http://plotdigitizer.sourceforge.net.

40. T. A. Worthington, P. S. E. Ermgassen, D. A. Friess, K. W. Krauss, C. E. Lovelock, J. Thorley, R. Tingey, C. D. Woodroffe, P. Bunting, N. Cormier, D. Lagomasino, R. Lucas, N. J. Murray, W. J. Sutherland, M. Spalding, A global biophysical typology of mangroves and its relevance for ecosystem structure and deforestation. *Sci. Rep.* **10**, 14652 (2020).

41. J. B. Kauffman, D. C. Donato, "Protocols for the measurement, monitoring and reporting of structure, biomass and carbon stocks in mangrove forests," Working papers 68, Cifor, Bogor Indonesia, 2012.

42. S. E. Hamilton, D. A. Friess, Global carbon stocks and potential emissions due to mangrove deforestation from 2000 to 2012. *Nat. Clim. Change* **8**, 240–244 (2018).

43. D. P. Rodrigues, C. Hamacher, G. C. D. Estrada, M. L. G. Soares, Variability of carbon content in mangrove species: Effect of species, compartments and tidal frequency. *Aquat. Bot.* **120**, 346–351 (2015).

44. M. F. Adame, S. Cherian, R. Reef, B. Stewart-Koster, Mangrove root biomass and the uncertainty of belowground carbon estimations. *For. Ecol. Manage.* **403**, 52–60 (2017).

45. A. S. Rovai, R. R. Twilley, E. Castaneda-Moya, P. Riul, M. Cifuentes-Jara, M. Manrow-Villalobos, P. A. Horta, J. C. Simonassi, A. L. Fonseca, P. R. Pagliosa, Global controls on carbon storage in mangrove soils. *Nat. Clim. Change* **8**, 534–538 (2018).

46. R. MacKenzie, S. Sharma, A. Rovai, "Environmental drivers of blue carbon burial and soil carbon stocks in mangrove forests" in *Dynamic Sedimentary Environments of Mangrove Coasts* (Elsevier, 2021), pp. 275–294.

47. I. C. Feller, D. F. Whigham, K. L. McKee, C. Lovelock, Nitrogen limitation of growth and nutrient dynamics in a disturbed mangrove forest, Indian River Lagoon, Florida. *Ecosyst. Ecol.* **134**, 405–414 (2003).

48. E. Castañeda-Moya, R. R. Twilley, V. H. Rivera-Monroy, B. D. Marx, C. Coronado-Molina, S. M. Ewe, Patterns of root dynamics in mangrove forests along environmental gradients in the Florida Coastal Everglades, USA. *Ecosystems* **14**, 1178–1195 (2011).

49. M. F. Adame, C. Teutli, N. S. Santini, J. P. Caamal, A. Zaldívar-Jiménez, R. Hernández, J. A. Herrera-Silveira, Root biomass and production of mangroves surrounding a karstic oligotrophic coastal lagoon. *Wetlands* **34**, 479–488 (2014).

50. M. A. Hayes, A. Jesse, B. Hawke, J. Baldock, B. Tabet, D. Lockington, C. E. Lovelock, Dynamics of sediment carbon stocks across intertidal wetland habitats of Moreton Bay, Australia, *Glob. Chang. Biol.* **23**, 4222–4234 (2017).

51. A. Gelman, A. Jakulin, M. G. Pittau, Y. S. Su, A weakly informative default prior distribution for logistic and other regression models. *Annal. Appl. Stat.* **2**, 1360–1383 (2008).

52. P. C. Bürkner, brms: An R Package for Bayesian multilevel models using Stan. *J. Stat. Softw.* **80**, 1–28 (2017).

53. P. C. Bürkner, Advanced Bayesian multilevel modeling with the R rackage brms. *R J.* **10**, 395–411 (2018).

54. R Core Team, R: A language and environment for statistical computing, version 4.1.1, R Foundation for Statistical Computing, Vienna, Austria (2021); www.R-project.org/.

55. A. S. Rovai, R. R. Twilley, E. Castañeda-Moya, S. R. Midway, D. A. Friess, C. C. Trettin, J. J. Bukoski, A. E. L. Stovall, P. R. Pagliosa, A. L. Fonseca, R. A. Mackenzie, A. Aslan, S. D. Sasmito, M. Sillanpää, T. G. Cole, J. Purbopuspito, M. W. Warren, D. Murdiyarso, W. Mofu, S. Sharma, P. H. Tinh, P. Riul, Mangrove forest structure dataset, version 1, Smithsonian data repository (2021); https://doi.org/10.6084/m9.figshare.13570601.v1.

56. Coastal Carbon Research Coordination Network (CCRN), Coastal wetland soil core profile data, CCRN Coastal Carbon Atlas (2023); https://doi.org/10.25573/serc.21565671

57. F. B. Golley, J. T. McGinnis, R. T. Clements, G. I. Child, M. J. Duever, *Mineral Cycling in a Tropical Moist Forest Ecosystem* (University of Georgia Press, 1975).

58. A. T. M. R. Hoque, S. Sharma, A. Hagihara, Above and belowground carbon acquisition of mangrove Kandelia obovata trees in Manko Wetland, Okinawa, Japan. *Int. J. Environ.* **1**, 7–13 (2011).

59. M. N. I. Khan, R. Suwa, A. Hagihara, Biomass and aboveground net primary production in a subtropical mangrove stand of Kandelia obovata (S., L.) Yong at Manko Wetland, Okinawa, Japan. *Wetl. Ecol. Manag.* **17**, 585–599 (2009).

60. A. Komiyama, K. Ogino, S. Aksornkoae, S. Sabhasri, Root biomass of a mangrove forest in southern Thailand. 1. Estimation by the trench method and the zonal structure of root biomass. *J. Trop. Ecol.* **3**, 97–108 (1987).

61. A. Komiyama, H. Moriya, S. Prawiroatmodjo, T. Toma, K. Ogino, "Primary productivity of mangrove forest" in *Biological System of Mangroves. A Report of East Indonesian Mangrove Expedition* (Ehime University, 1988), pp. 97–117.

62. A. Komiyama, S. Havanond, W. Srisawatt, Y. Mochida, K. Fujimoto, T. Ohnishi, S. Ishihara, T. Miyagi, Top/root biomass ratio of a secondary mangrove (*Ceriops tagal* (Perr.) C. B. Rob.) forest *Forest Ecol. Manag.* **139**, 127–134 (2000).

63. A. P. Mackey, Biomass of the mangrove *Avicennia marina* (Forsk.) Vierh. near Brisbane, south-eastern Queensland. *Mar. Freshw. Res.* **44**, 721–725 (1993).

64. T. Mori, R. Tabuchi, K. Fujimoto, H. Utsugi, S. Kuramoto, M. Hiraide, A. Imaya, " US-Japan joint research for conservation and management of mangrove forests in the South Pacific Islands–Dynamics and production of mangrove forests in Pohnpei Island" (Report of the Bilateral International Joint Research, Forestry and Forest Products Research Institute, Japan 1997).

65. R. Ray, D. Ganguly, C. Chowdhury, M. Dey, S. Das, M. K. Dutta, S. K. Mandal, N. Majumder, T. K. Jana, Carbon sequestration and annual increase of carbon stock in a mangrove forest. *Atmos. Environ.* **45**, 5016–5024 (2011).

66. D. M. Alongi, F. Tirendi, B. F. Clough, Below-ground decomposition of organic matter in forests of the mangroves *Rhizophora stylosa* and *Avicennia marina* along the arid coast of Western Australia. *Aquat. Bot.* **68**, 97–122 (2000).

67. A. I. Robertson, D. M. Alongi, Massive turnover rates of fine root detrital carbon in tropical Australian mangroves. *Oecologia* **180**, 841–851 (2016).

68. N. Saintilan, Above- and below-ground biomass of mangroves in a sub-tropical estuary. *Mar. Freshw. Res.* **48**, 601–604 (1997).

69. N. Saintilan, Above- and below-ground biomasses of two species of mangrove on the Hawkesbury River estuary, New South Wales. *Mar. Freshw. Res.* **48**, 147–152 (1997).

70. D. Santos, G. C. Estrada, V. Fernandez, M. R. Estevam, B. T. Souza, M. L. Soares, First assessment of carbon stock in the belowground biomass of Brazilian mangroves. *An. Acad. Bras. Cienc.* **89**, 1579–1589 (2017).

71. R. E. Sherman, T. J. Fahey, P. Martinez, Spatial patterns of biomass and aboveground net primary productivity in a mangrove ecosystem in the Dominican Republic. *Ecosystems* **6**, 384–398 (2003).

72. N. F. Y. Tam, Y. S. Wong, C. Y. Lan, G. Z. Chen, Community structure and standing crop biomass of a mangrove forest in Futian Nature Reserve, Shenzhen, China, *Hydrobiologia* **295**, 193–201 (1995).

73. F. Tamooh, M. Huxham, M. Karachi, M. Mencuccini, J. G. Kairo, B. Kirui, Below-ground root yield and distribution in natural and replanted mangrove forests at Gazi bay, Kenya. *Forest Ecol. Manag.* **256**, 1290–1297 (2008).

74. P. Tran, I. Gritcan, J. Cusens, A. C. Alfaro, S. Leuzinger, Biomass and nutrient composition of temperate mangroves (*Avicennia* marinavar. australasica) in New Zealand. *N. Z. J. Mar. Freshw. Res.* **51**, 427–442 (2017).

75. K. Fiala, L. Hernández, P. Holub, Comparison of vertical distribution of live and. *J. Tropical Forest Sci.* **29**, 275–281 (2017).

76. S. V. Briggs, Estimates of biomass in a temperate mangrove community. *Aust. J. Ecol.* **2**, 369–373 (1977).

77. E. Castaneda-Moya, "Landscape patterns of community structure, biomass and net primary productivity of mangrove forests in the Florida Coastal Everglades as a function of resource, regulators, hydroperiod, and hurricane disturbance," thesis, Louisiana State University and Agricultural and Mechanical College (2010).

78. B. Chalermchatwilai, S. Poungparn, P. Patanaponpaiboon, Distribution of fine-root necromass in a secondary mangrove forest in Trat province, Eastern Thailand, *ScienceAsia* **37**, 1–5 (2011).

79. N. Cormier, R. R. Twilley, K. C. Ewel, K. W. Krauss, Fine root productivity varies along nitrogen and phosphorus gradients in high-rainfall mangrove forests of Micronesia. *Hydrobiologia* **750**, 69–87 (2015).

80. K. Fiala, L. Hernández, Root biomass of a mangrove forest in southwestern Cuba (Majana). *Ecology (Bratislava)* **12**, 15–30 (1993).

81. S. M. Gleason, K. C. Ewel, Organic matter dynamics on the forest floor of a Micronesian mangrove forest: An investigation of species composition Shifts1. *Biotropica* **34**, 190–198 (2002).

82. F. B. Golley, H. T. Odum, R. F. Wilson, The structure and metabolism of a Puerto Rican red mangrove forest in May. *Ecology* **43**, 9–19 (1962).

83. K. Lamont, N. Saintilan, J. J. Kelleway, D. Mazumder, A. Zawadzki, Thirty-year repeat measures of mangrove Above- and below-ground biomass reveals unexpectedly high carbon sequestration. *Ecosystems* **23**, 370–382 (2020).

84. H. H. Checon, G. N. Corte, C. F. Silva, Y. Schaeffer-Novelli, A. C. Z. Amaral, Mangrove vegetation decreases density but does not affect species richness and trophic structure of intertidal polychaete assemblages. *Hydrobiologia* **795**, 169–179 (2017).

85. Y. Schaeffer-Novelli, G. Cintrón-Molero, A. S. Reis-Neto, G. M. Abuchahla, L. C. Neta, C. F. Lira-Medeiros, The mangroves of Araçá Bay through time: An interdisciplinary approach for conservation of spatial diversity at large scale. *Ocean Coast. Manag.* **164**, 60–67 (2018).

86. A. P. L. M. Madi, M. R. T. Boeger, C. B. Reissmann, Description of the soil and root biomass of two subtropical mangroves in Antonina and Guaratuba Bay, Paraná State, Brazil, *Hoehnea* **44**, 328–335 (2017).

87. Y. Xiong, X. Liu, W. Guan, B. Liao, Y. Chen, M. Li, C. Zhong, Fine root functional group based estimates of fine root production and turnover rate in natural mangrove forests. *Plant Soil* **413**, 83–95 (2017).

88. Z. He, Y. Peng, D. Guan, Z. Hu, Y. Chen, S. Y. Lee, Appearance can be deceptive: Shrubby native mangrove species contributes more to soil carbon sequestration than fast-growing exotic species. *Plant Soil* **432**, 425–436 (2018).

89. S. M. Muhammad-Nor, M. Huxham, Y. Salmon, S. J. Duddy, A. Mazars-Simon, M. Mencuccini, P. Meir, G. Jackson, Exceptionally high mangrove root production rates in the

Kelantan Delta, Malaysia; An experimental and comparative study. *For. Ecol. Manage.* **444**, 214–224 (2019).

90. J. R. Torres, E. Barba, F. J. Choix, Production and biomass of mangrove roots in relation to hydroperiod and physico-chemical properties of sediment and water in the Mecoacan Lagoon, Gulf of Mexico. *Wetl. Ecol. Manag.* **27**, 427–442 (2019).

91. R. H. Bulmer, L. Schwendenmann, C. J. Lundquist, Carbon and nitrogen stocks and below-ground allometry in temperate mangroves. *Front. Mar. Sci.* **3**, 150 (2016).

92. D. H. Phillips, M. P. Kumara, L. P. Jayatissa, K. W. Krauss, M. Huxham, Impacts of mangrove density on surface sediment accretion, belowground biomass and biogeochemistry in Puttalam Lagoon, Sri Lanka, *Wetlands* **37**, 471–483 (2017).

93. M. A. Njana, O. M. Bollandsås, T. Eid, E. Zahabu, R. E. Malimbwi, Above- and belowground tree biomass models for three mangrove species in Tanzania: A nonlinear mixed effects modelling approach. *Annal. Forest. Sci.* **73**, 353–369 (2016).

94. E. S. Yando, M. J. Osland, M. W. Hester, Microspatial ecotone dynamics at a shifting range limit: Plant–soil variation across salt marsh–mangrove interfaces. *Oecologia* **187**, 319–331 (2018).
